# HDAC2 inhibits EMT-mediated cancer metastasis by downregulating the long noncoding RNA H19 in colorectal cancer

**DOI:** 10.1186/s13046-020-01783-9

**Published:** 2020-12-02

**Authors:** Xue-ting Hu, Wei Xing, Rong-sen Zhao, Yan Tan, Xiao-feng Wu, Luo-quan Ao, Zhan Li, Meng-wei Yao, Mu Yuan, Wei Guo, Shang-ze Li, Jian Yu, Xiang Ao, Xiang Xu

**Affiliations:** 1 Department of Stem Cell & Regenerative Medicine, State Key Laboratory of Trauma, Burn and Combined Injury, Daping Hospital, Army Medical University, Chongqing, 400042 People’s Republic of China; 2 Department of Biochemistry and Molecular Biology, College of Basic Medical Science, Army Medical University, Chongqing, 400042 People’s Republic of China; 3 Medical Science Research Center, Zhongnan Hospital of Wuhan University, Wuhan, 430071 People’s Republic of China; 4 Department of Pathology, University of Pittsburgh, UPMC Hillman Cancer Center, Pittsburgh, PA 15213 USA

**Keywords:** CRC, Metastasis, HDAC2, H19, EMT

## Abstract

**Background:**

Emerging evidence suggests that epithelial mesenchymal transition (EMT) and epigenetic mechanisms promote metastasis**.** Histone deacetylases (HDACs) and noncoding RNAs (ncRNAs) are important epigenetic regulators. Here, we elucidated a novel role of histone deacetylase 2 (*HDAC2*) in regulating EMT and CRC metastasis via ncRNA.

**Methods:**

The expression of HDACs in CRC was analyzed using the public databases and matched primary and metastatic tissues, and CRC cells with different metastatic potentials (DLD1, HCT116, SW480 and SW620). Microarray analysis was used to identify differential genes in parental and *HDAC2* knockout CRC cells. EMT and histone modifications were determined using western blot and immunofluorescence. Migration ability was assessed by transwell assay, and metastasis was assessed in vivo using a tail vain injection. Gene expression and regulation was assessed by RT-PCR, chromatin immunoprecipitation and reporter assays. Protein interaction was assessed by immunoprecipitation. Specific siRNAs targeting *H19*, *SP1* and *MMP14* were used to validate their role in *HDAC2* loss induced EMT and metastasis.

**Results:**

Reduced HDAC2 expression was associated with poor prognosis in CRC patients and found in CRC metastasis. *HDAC2* deletion or knockdown induced EMT and metastasis by upregulating the long noncoding RNA *H19* (*LncRNA H19*). HDAC2 inhibited LncRNA H19 expression by histone H3K27 deacetylation in its promoter via binding with SP1. LncRNA H19 functioned as a *miR-22-3P* sponge to increase the expression of MMP14. *HDAC2* loss strongly promoted CRC lung metastasis, which was suppressed *LncRNA H19* knockdown.

**Conclusion:**

Our study supports HDAC2 as a CRC metastasis suppressor through the inhibition of EMT and the expression of H19 and MMP14.

**Supplementary Information:**

The online version contains supplementary material available at 10.1186/s13046-020-01783-9.

## Background

Colorectal cancer (CRC) is one of the most common malignancies worldwide [1]. Despite the progress in early diagnosis and therapy in the past decade, the overall survival of CRC patients remains low in the setting of metastasis [2]. Metastasis is a complex process, and many cell-intrinsic identities and extrinsic microenvironment factors influence the metastatic potential of CRC cells [3]. A better mechanistic understanding of metastasis may provide novel therapeutic targets to help improve the overall survival of CRC patients.

Epithelial to mesenchymal transition (EMT) is characterized by the loss of cell-cell adhesion and the gain of migratory and invasive traits [4]. EMT has been reported to play important roles in many physiological and pathological processes [5]. EMT frequently occurs at the initial stage of cancer metastasis. During EMT, epithelial cells evade extracellular constraints from adhesion molecules such as E-cadherin. Meanwhile, epithelial cells upregulate the expression of mesenchymal markers, such as fibronectin and ITGα5, to increase cell motility [6]. EMT and related molecules have been suggested as potential prognostic or therapeutic targets in cancer [7]. However, mechanistic understanding of EMT in metastasis is still limited.

Histone deacetylases (HDACs) are enzymes that catalyze the removal of acetyl groups from lysine residues in histones to suppress gene transcription. HDACs also have non-histone substrates. Eighteen classes of HDACs have been identified in mammals [8], and are categorized as HDAC1–11 and SIRT1–7. Deregulated expression of HDACs in cancer has been extensively documented. Aberrant expression of classical (class I, II, IV) HDACs has been linked to the initiation and progression of a variety of cancers [8]. In most cases, a higher level of HDACs is associated with advanced disease and poor outcome in tumor patients, presumably due to the loss of tumor suppressor functions [9–11]. However, mutations and reduced expression of HDACs have also been reported in cancer. *HDAC1* somatic mutations were detected in 8.3% of dedifferentiated liposarcomas, and *HDAC4* homozygous deletions occurred in 4% of melanoma [12, 13]. Low HDAC10 expression was associated with poor prognosis in lung and gastric cancer patients [14, 15]. HDAC6 was downregulated in human hepatocellular carcinoma (HCC) tissues, and it down regulation was associated with poor prognosis in liver transplantation patients. *HDAC6* knockdown promoted angiogenesis in HCC [16]. Truncating mutations and the loss of HDAC2 protein expression have been observed in human epithelial cancers with microsatellite instability [17]. Furthermore, the ectopic expression of HDAC2 in *HDAC2* mutant cancer cells inhibited tumor cell growth in vitro and in vivo [18]. Collectively, these findings support potential tumor suppressive functions of HDACs in cancer initiation and maintenance.

The roles of HDACs in EMT and cancer metastasis are context-dependent. HDAC1 promoted EMT in gallbladder cancer by binding with TCF12 [19]. HDAC1 and HDAC2 were recruited by the transcriptional repressor ZEB1 to downregulate E-cadherin expression in pancreatic cancer [20]. SIRT1 induced EMT and enhanced prostate cancer cell migration and metastasis by cooperating with ZEB1 [21]. HDAC inhibitors (HDACis) have been developed as potential anticancer agents, and can restore the expression and function of tumor suppressors [8]. However, HDACis also promote tumor progression, EMT and cancer metastasis in some models [22]. Cell migration was dramatically enhanced by various classes of HDACis in 13 of 30 human breast, gastric, liver, and lung cancer cell lines examined in a dose-dependent manner [23]. Metastasis was also enhanced in HDACi-treated mice through the activation of multiple PKCs and downstream substrates along with upregulated p21 expression [23]. Nonselective HDACis such as Trichostatin A (TSA) and valproic acid (VPA) induced mesenchymal features in colon carcinoma cells with decreased expression in E-cadherin and increased expression in vimentin [24].

In this study, we found that reduced expression of HDAC2 in CRC metastasis is associated with poor patient survival. *HDAC2* deletion in CRC cells increased EMT-mediated metastasis in vivo and in vitro. Mechanistically, HDAC2 suppressed EMT and CRC metastasis through the inhibition of the H19/MMP14 axis. Taken together, these results establish HDAC2 as a novel metastasis suppressor in CRC.

## Materials and methods

### Dataset

The relative expression data of HDAC1, HDAC2, HDAC3 and HDAC8 were downloaded from the Oncomine public database (www.oncomine.org). The dataset contains 330 primary sites and 43 metastatic CRC tissue samples.

### Survival analysis of HDAC1, HDAC2, HDAC3 and HDAC8

We labeled TCGA samples as “high” or “low” according to whether the expression of HDAC1, HDAC2, HDAC3 and HDAC8 was higher or lower than the corresponding median value among all samples. The log-rank test was used to measure whether the survival time was significantly different between the “high” and “low” expression groups. Kaplan-Meier plots were made by Gene Expression Profiling Interactive Analysis (GEPIA: http://gepia.cancer-pku.cn/).

### CRC tissue sample and immunochemistry

Commercially available tissue microarray (TMA) slides (HLin-Ade075Met-01, Shanghai Biochip Co., Ltd., Shanghai, China) containing histologically confirmed tissues from CRC patients were purchased for immunohistochemistry (IHC) analysis. Specific primary antibodies against HDAC2 (Cell Signaling Technology) were used for IHC with a 2-step protocol.

### Cell culture

DLD1, HCT116, SW480 and SW620 cells were obtained from ATCC. DLD1 and HCT116 cells were cultured in RPMI 1640 medium, while SW480 and SW620 were cultured in high glucose DMEM. All media were supplemented with 10% FBS, 100 μg/m L penicillin and 100 U/m L streptomycin. The DLD1 *HDAC2* KO cell line was constructed in the lab of Professor Run-lei Du, who is our collaborator in this study [25].

### Microarray analysis

The total RNA from DLD1 and DLD1 *HDAC2* KO cells was prepared for microarray analysis (*n* = 3 each). The Affymetrix microarray was used to detect mRNA and long noncoding RNA expression profiles. Microarray data were normalized using the RAM (robust multiple-array average) normalization method. The differentially expressed genes were determined with a threshold cutoff of 2-fold (*p* < 0.01).

### Transwell migration assays

Tumor cell migration assays were performed according to the manufacturer’s instructions. Briefly, cells were harvested and resuspended in serum-free medium and then seeded onto Transwell inserts at a density of 100,000 cells/well. Then, the inserts were placed in a lower chamber filled with 600 μl culture media containing 10% FBS. Transwells were incubated for 24 h at 37 °C. Cells on the inside of the Transwell inserts were removed with a cotton swab; then, cells that migrated to the lower surface of the membrane were fixed with 4% paraformaldehyde and stained with 0.1% crystal violet. Photographs were taken from five random fields, and the cells were counted to calculate the average number of cells that had transmigrated.

### Vector construction and luciferase reporter assay

The *H19* promoter containing intact SP1 recognition sequences was PCR-amplified and subcloned into the KpnI and HindIII sites of the pGL-3-basic vector, and the vector was named pGL-3-H19. The pGL3-H19 vector with point mutations in the SP1 binding sites was synthesized by GenScript (Nanjing, China) and named pGL3-H19-mut (SP1). For the luciferase reporter assay, HEK293 cells cultured in 24-well plates were cotransfected with luciferase reporter plasmids and HDAC2 plasmids. Twenty-four hours posttransfection, HEK293 cells were lysed in lysis buffer. After centrifugation at 12,000 rpm for 5 min, the supernatant was transferred to a new tube. The luciferase activity was monitored by mixing 10 μl supernatant with 30 μl luciferase assay buffer and using a GloMax 20/20 Luminometer (Promega).

### siRNA and gene transfection

The siRNAs were synthesized by RiboBio Company (Guangzhou, China). Oligonucleotide transfection was conducted by using Lipofectamine™ RNAiMAX transfection reagent (Life, USA) following the protocol recommended by the manufacturer. After 48 h posttransfection, the cells were collected and used for further investigations.

### Chromatin immunoprecipitation (ChIP) and q PCR

ChIP was performed using a SimpleChIP® Enzymatic Chromatin IP Kit (CST) following the manufacturer’s instructions. Briefly, genomic DNA-protein complexes were immunoprecipitated using anti-HDAC2 antibody or normal rabbit IgG as a control. After enzyme digestion and sonication, the precipitated DNA was amplified by SYBR Green-based quantitative real-time PCR using primers encompassing the promoter regions of the *H19* gene. The ChIP PCR primers used were (the numbers in parentheses indicate the sequence regions corresponding to GenBank ID AF125183):

- Primer 1: 5′-CCAGCCATGTGCAAAGTATG-3′ (9747–9766)
- Primer 2: 5′-CCATCCTGGAATTCTCCAAA-3′ (9939–9920)
- Primer 3: 5′-GCGGTCTTCAGACAGGAAAG-3′ (9468–9487)
- Primer 4: 5′-CACGTTCCTGGAGAGTAGGG-3′ (9673–9654)

### Co-immunoprecipitation

A co-immunoprecipitation assay was performed in the following steps. Briefly, the cells were washed with ice-cold PBS, lysed in NP-40 buffer containing cocktail and then centrifuged for 10 min at 12000 rpm and 4 °C. Anti-SP1/HDAC2 antibody or normal rabbit IgG was added to the cell lysate and incubated at 4 °C overnight. Then, 15 μl of protein A/G agarose beads was added to each tube, incubated at room temperature for 3 h and centrifuged for 3 min at 4000 rpm at 4 °C. A total of 30 μl of 2× SDS-loading buffer was added to the antigen-antibody-protein A/G agarose bead complex, which was boiled for 10 min. The sample was collected for subsequent SDS-PAGE and Western blotting.

### RNA-binding protein immunoprecipitation

The anti-Ago2 RIP assay was performed using a Magan RIP™ RNA-Binding protein Immunoprecipitation Kit (Milipore) following the manufacturer’s instructions. Briefly, DLD1 *HDAC2* KO cells were washed with cold PBS and lysis by RIP Lysis Buffer. After that, the cell lysates were incubated with antibody against Ago2 (Milipore, USA). The normal Mouse IgG was used as negative control. For RNA immunoprecipitation, the supernatant was incubated with the antibody-coated Sepharose beads overnight. The RNA bound to Ago2 antibody was extracted with TRIzol reagent (Invitrogen, USA) and detected by qRT-PCR.

### Fluorescence in situ hybridization (FISH)

The sequence of the H19 probe was 5′-FAM/GCTGCTGTTCCGATGGTGTCTTTGATGTTGGGC/FAM-3′; this probe was used for FISH of H19 from B-NDG mouse pulmonary metastases. After the metastatic lung tissues were removed and cleaned, they were immediately fixed in an ISH fixation solution (DEPC water preparation) for 12 h. After tissue fixation, they were dehydrated by gradient alcohol and then embedded in paraffin. The tissue sections were used for H19 probe hybridization, and the nuclei were restained by DAPI. The sections were observed under a fluorescence microscope, and images were collected. The H19 probe is shown in green, while DAPI staining is shown in blue.

### Animal study

B-NDG immunodeficient mice (with T cell, B cell and NK cell defects) were obtained from Beijing Biocytogen Biotechnology Co., Ltd. The B-NDG mice were randomly divided into two groups. A total of 5 **×** 10^6^ luciferase-labeled CRC cells were injected intravenously into B-NDG mice via the tail vein. Four weeks later, the mice were anesthetized and injected intraperitoneally with fluorescein potassium salt (150 mg/kg), and 10 min later, the metastatic tumor was detected and photographed by a bioluminescent in vivo imager (VILBER Fusion FX7, France). The mice were sacrificed, and their lung tissues were removed for H&E staining, immunohistochemistry and FISH. The mean number of metastatic nodules in B-NDG mice with lung metastasis was calculated.

### Statistical analysis

All experiments were repeated no fewer than 3 times. Experimental results are presented as the mean ± S.E.M. The statistical significance of comparisons between two groups was determined with a two-tailed Student’s t-test. *P*<0.05 indicated statistical significance.

### Additional materials and methods

More details regarding the materials and methods can be found in the supplementary information.

## Results

### HDAC2 is frequently downregulated in metastatic colorectal cancer tissues

**Fig. 1:**
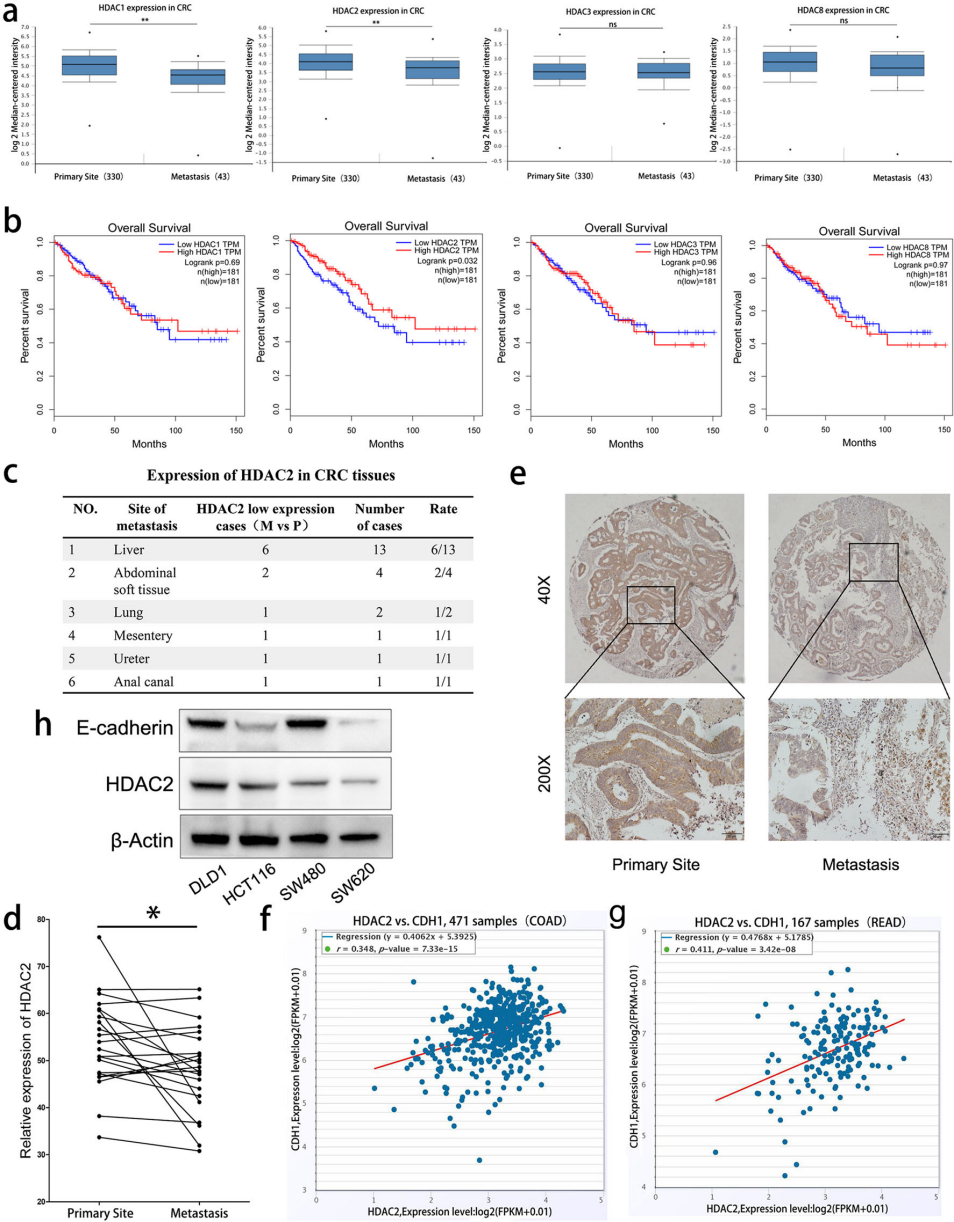
HDAC2 is frequently downregulated in human metastatic colorectal tissues. **a** Relative expression of HDAC1, HDAC2, HDAC3, and HDAC8 in CRC tissues. Data were collected from Oncomine, including 330 cases of primary site and 43 cases of metastasis. **b** Kaplan-Meier analyses of the correlations between HDAC1, HDAC2, HDAC3, and HDAC8 expression levels and overall survival in 362 patients with CRC. Data were collected from TCGA. We labeled TCGA samples as “high” or “low” according to whether the expression of HDAC1, HDAC2, HDAC3 and HDAC8 was higher or lower than the corresponding median value among all samples. **c**, **d** Expression of HDAC2 in 22 paired CRC samples. **e** Representative immunohistochemical staining of a tissue array containing CRC samples with anti-HDAC2 antibody. Magnification: 40× (upper) and 200× (lower). **f, g** Expression correlations between HDAC2 and E-cadherin in CRC samples. Data were collected from TCGA, including 471 COAD samples and 167 READ samples. **h** Expression of E-cadherin and HDAC2 in CRC cell lines detected by Western Blot. **P < 0 .01; *P < 0.05. The data are representatives and are presented as mean ± standard error of the mean of 3 assays

To investigate the roles of HDACs in colorectal cancer metastasis, we queried their expression in the public database Oncomine (www.oncomine.org). The results showed that, compared with primary tumors, the expression of HDAC1 and HDAC2, but not HDAC3 and HDAC8 is significantly lower in metastases (Fig. 1a). Notably, all of HDACs (1–3, 8, or), Kaplan-Meier analyses indicated that only low expression of HDAC2 is correlated with poor survival of CRC patients (Fig. 1b). The expression of HDAC2 was detected in 22 paired CRC specimens containing matched primary and metastatic tissues samples by IHC (Fig. 1e and Fig. S1a). The results showed that the expression of HDAC2 is significantly lower in 12 cases (M vs P) (Fig. 1c and d). Mining TCGA database using analyzing tools from starBase database (http://starbase.sysu.edu.cn/),we found that the expression of CDH1 (E-cadherin), a well-accepted marker negatively associated with EMT and metastasis, is positively correlated with the expression of HDAC2 in CRC(including 471 COAD samples and 167 READ samples, Fig. 1f and g). In addition, the expression of HDAC2 displays a lower level in highly invasive CRC cells (HCT116 and SW620) (Fig. 1h and Fig. S1b, c). Collectively, these findings indicate that HDAC2 is significantly downregulated in metastatic CRC tissues, which is associated with poor prognosis in CRC patients.

### Low HDAC2 expression induces EMT in CRC cells

**Fig. 2:**
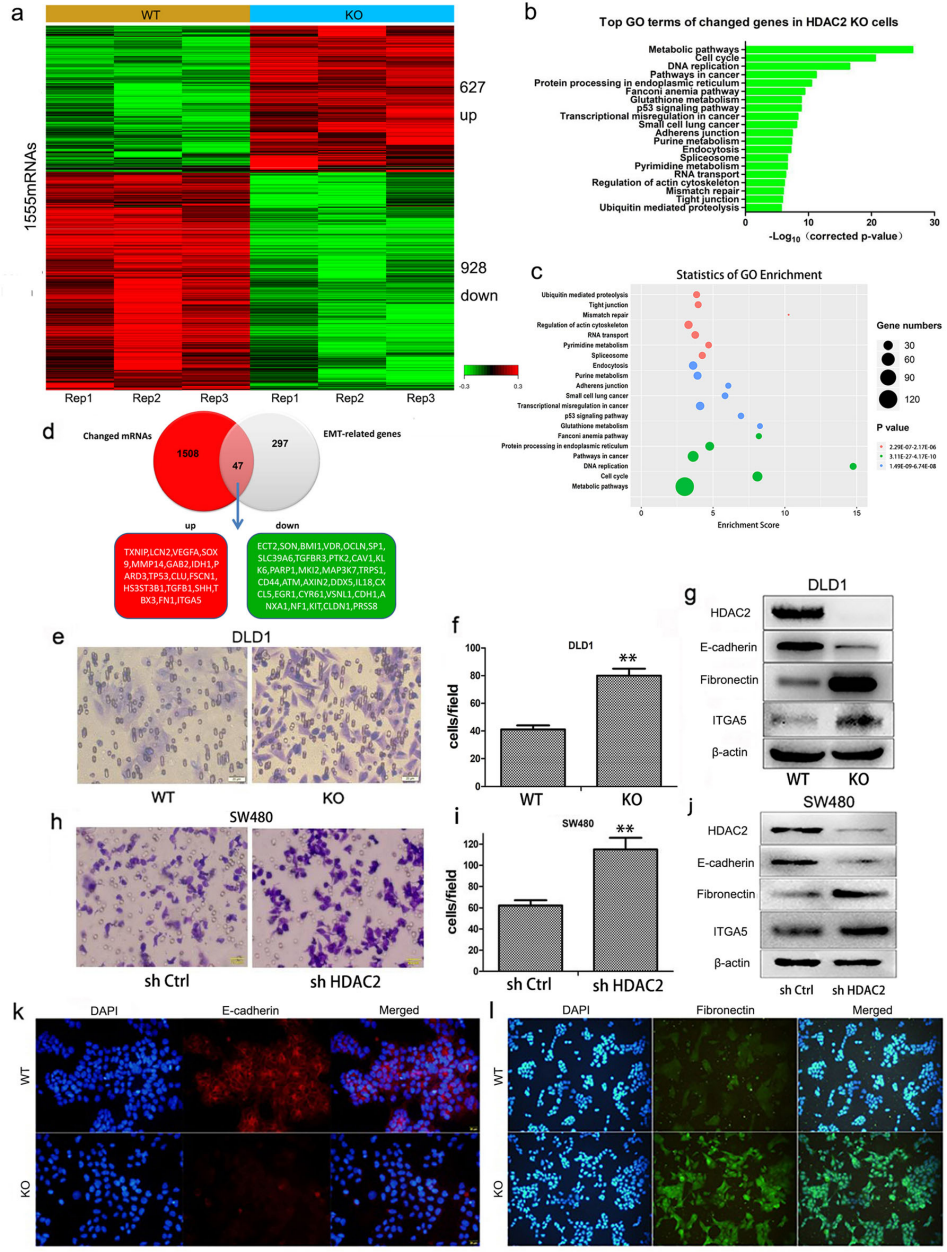
Reduced HDAC2 induces EMT in CRC. **a** Altered mRNA expression in DLD1 *HDAC2* KO cells. **b**, **c** Pathway analysis of changed genes in DLD1 HDAC2 KO cells (compared with DLD1 WT cells). **d** Merged mRNAs for EMT-related genes and those identified in our microarray. **e**, **f** Migration ability of DLD1 *HDAC2* KO cells detected by transwell. **g**, **k**, **l** EMT markers were analyzed by immunoblotting and IF with the indicated antibodies in DLD1 *HDAC2* WT and KO cells. **h**, **i** Migration ability of SW480 *HDAC2* RNAi cells detected by transwell. **j** EMT markers were analyzed by immunoblotting with the indicated antibodies in SW480 *HDAC2* RNAi cells. **P < 0 .01; *P < 0.05. The data are representatives and are presented as mean ± standard error of the mean of 3 assays

To explore whether HDAC2 plays a role in suppressing CRC metastasis, we knocked out *HDAC2* in DLD1 cells as previously described [25]. We then used microarray analysis to detect the differential expression of genes in *HDAC2* KO cells and found 1555 differential expression of mRNAs consisting of 627 upregulated genes and 928 downregulated genes (Fig. 2a and Supplementary Table 1). Overlap of the 1555 mRNAs and 344 genes involved in EMT (non-coding RNA was excluded, downloaded from http://dbemt.bioinfo-minzhao.org/dbemt1/index.html), 47 mRNAs were detected (Fig. 2d). Among those 47 mRNAs, we found that the epithelial marker CDH1 is downregulated, while the mesenchymal markers fibronectin and ITGA5 are upregulated (Fig. 2d). Pathway analysis of deregulated genes in KO cells showed strong enrichment of genes involved in pathways crucial for cancer metastasis, such as adherens junction and tight junction (Fig. 2b and c). These results indicated that HDAC2 modulates the expression profiling of EMT and metastasis related genes.

Moreover, we found that *HDAC2* deletion increases the conversion of epithelial to mesenchymal features in DLD1 cells. As shown in Figure S2a, DLD1 *HDAC2* KO cells display a non-polarized and spindle-shaped morphology. Consistent with these morphological changes, decreases in E-cadherin and increases in fibronectin and ITGA5 were observed at the protein level (Fig. 2g, k and l). In addition, we found that the migratory ability of KO cells is increased (Fig. 2e and f). The increased migratory ability of DLD1 KO cells were confirmed by Real Time Cellular Analysis (RTCA) (Fig. S2b). We found that *HDAC2* knockdown in SW480 also increases the conversion of epithelial to mesenchymal features, including increased migratory ability (Fig. 2h and i) and up-regulated fibronectin and ITGA5 (Fig. 2j). Collectively, these data show that HDAC2 negatively regulates EMT and CRC cell migration.

### Low HDAC2 expression induces EMT by upregulating the lncRNA H19

**Fig. 3:**
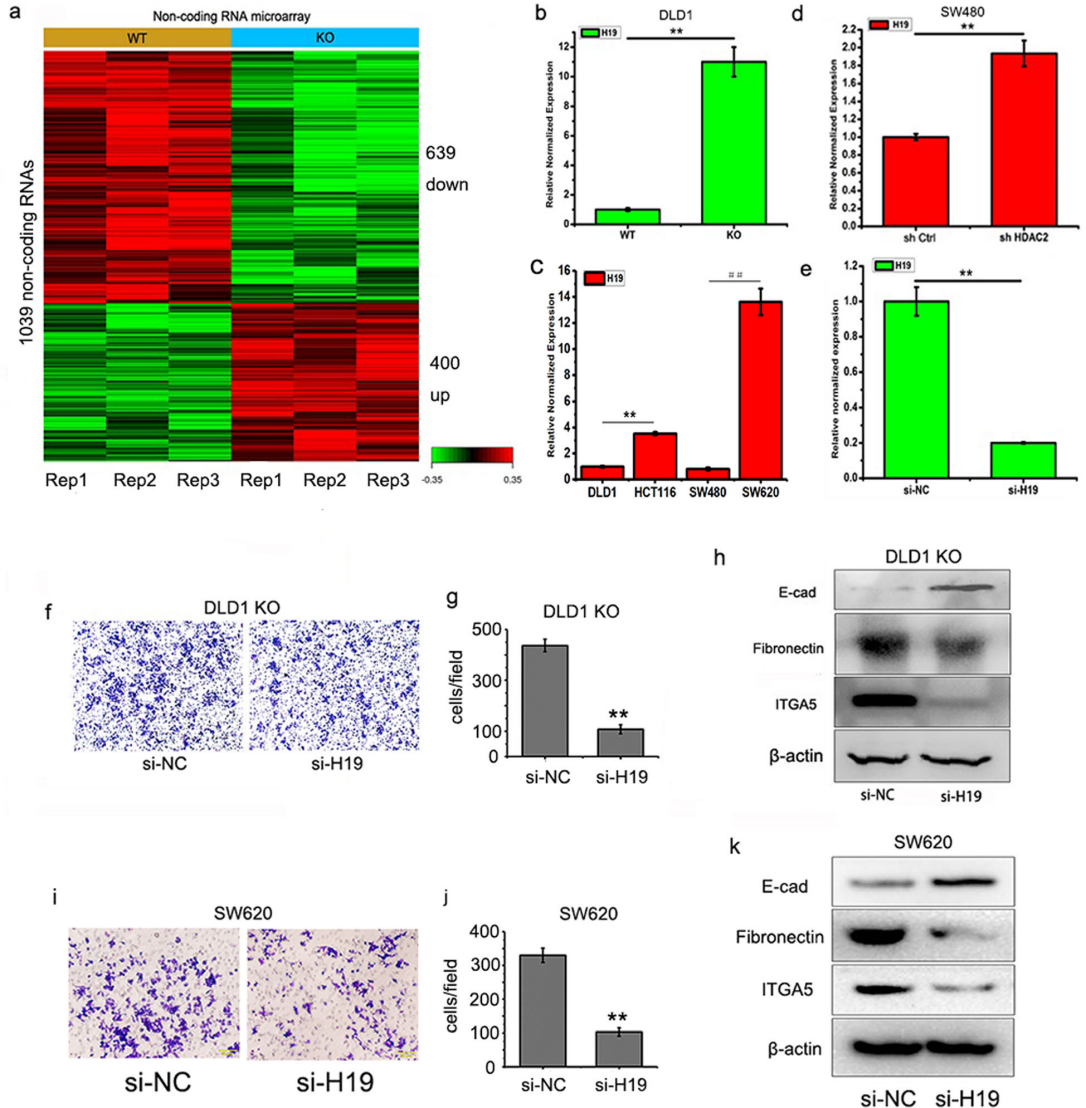
Reduced HDAC2 induces EMT in CRC by upregulating H19. **a** Altered non-coding RNAs expression in DLD1 *HDAC2* KO cells. **b** Expression of H19 in DLD1 *HDAC2* KO cells. **c** Expression of H19 in CRC cells. **d** Expression of H19 in SW480 *HDAC2* RNAi cells. **e**, **f**, **g** Migration ability of DLD1 *HDAC2* KO H19 RNAi cells detected by transwell. **h** Detection of EMT markers by Western Blot in DLD1 *HDAC2* KO H19 RNAi cells. **i**, **j** Migration ability of SW620 H19 RNAi cells detected by transwell. **h** Detection of EMT markers by Western Blot in SW620 *H19* RNAi cells. **P < 0 .01; *P < 0.05. The data are representatives and are presented as mean ± standard error of the mean of 3 assays

Noncoding RNAs have been reported to be important regulators for many biological processes. Recently, a series of noncoding RNAs were found to modulate the expression of genes involved in EMT and metastasis [26]. To understand the mechanism of HDAC2 in regulating EMT, we used microarray analysis to evaluate expression changes of noncoding RNAs in DLD1 *HDAC2* KO cells. Transcriptome profiling identified 1039 ncRNA transcripts with differentially expression in *HDAC2* KO cells (Fig. 3a and Supplementary Table 2). Among the most differentially expressed noncoding RNAs, a novel lncRNA (accession Number: n333410) drew our attention. The sequence of n333410 was downloaded from the NONCODE database (http://www.noncode.org/) and is fully aligned with *H19* (GenBank: AF125183.1) (Fig. S3a). Q-PCR assays using gene-specific primers for *H19* confirmed that the lncRNA *H19* is significantly upregulated in DLD1 *HDAC2* KO cells (Fig. 3b). *HDAC2* knockdown in SW480 cells by shRNA also increases the expression of *H19* (Fig. 3c). Moreover, the expression of *H19* in colorectal cancer cells with low HDAC2 expression is higher than that in cells with high HDAC2 expression (Fig. 3c). These findings indicate that HDAC2 is a negative regulator of H19 expression in CRC.

H19 was previously reported to be an important regulator in metastasis [27, 28]. We hypothesized that H19 is a downstream target of HDAC2 regulating EMT in CRC. To test our hypothesis, we synthesized and identified siRNA targeting *H19* (Fig. 3e) and transfected it into DLD1 *HDAC2* KO and SW620 cells. The results showed that the EMT is reversed in the DLD1 *HDAC2* KO and SW620 cells. *H19* knockdown by siRNA significantly reduces the migration ability of KO (Fig. 3f, g and S3b) and SW620 cells (Fig. 3i and j). Additionally, an increased E-cadherin and a decreased fibronectin and ITGA5 were observed after *H19* knockdown (Fig. 3h and k). Collectively, these findings support H19 as a downstream target of HDAC2 and EMT inducer in CRC.

### HDAC2 inhibits the expression of H19 through SP1-dependent H3K27 deacetylation

**Fig. 4:**
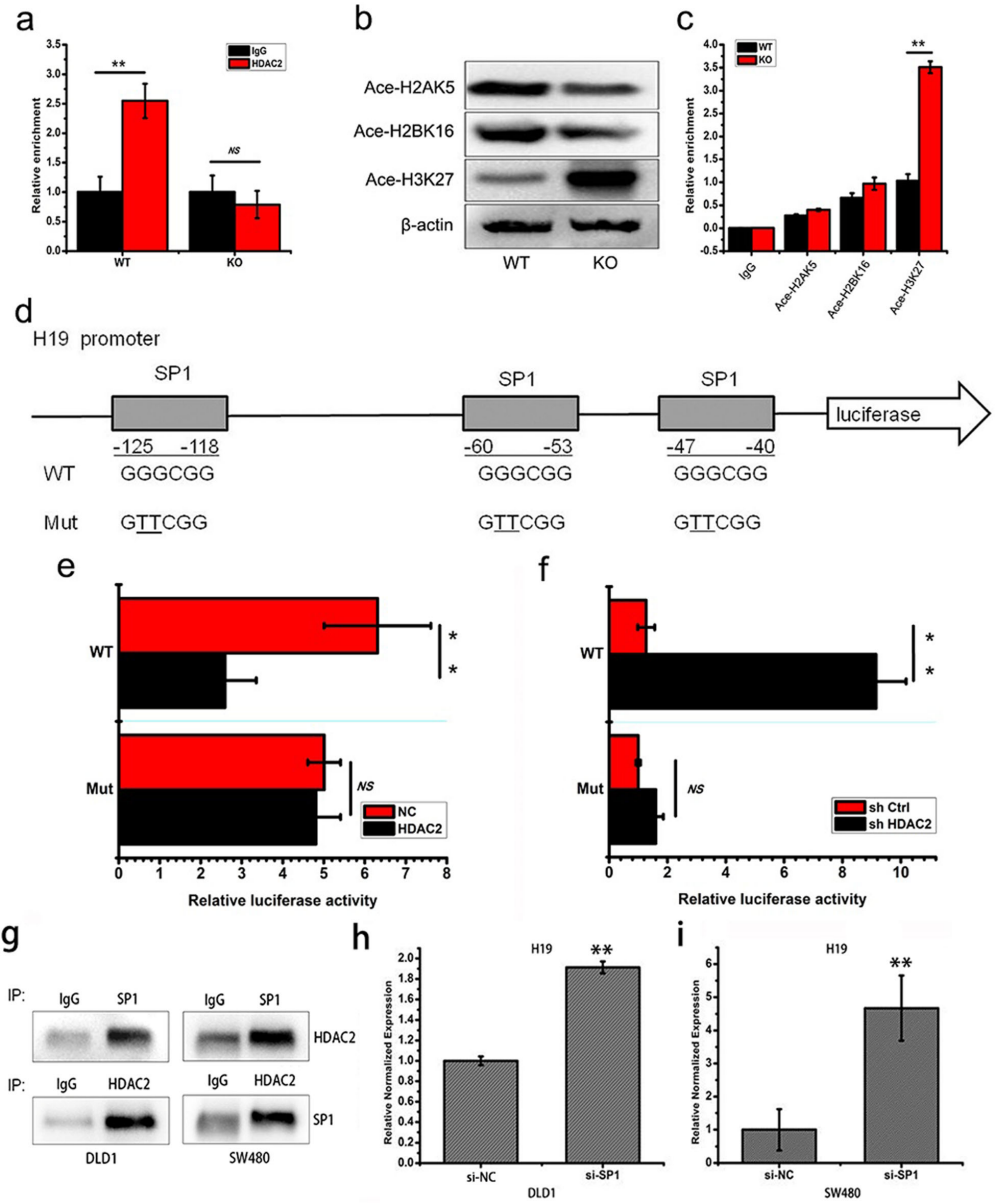
HDAC2 inhibits the expression of H19 by interacting with the transcription factor SP1 and catalyzing H3K27 deacetylation. **a** ChIP analysis of HDAC2 enrichment in the promoter of *H19* gene in DLD1 WT or *HDAC2* KO cells. **b** Detection of acetylated histone H2AK5, H2BK16 and H3K27 in DLD1 WT or *HDAC2* KO cells. **c** ChIP analysis of acetylated histone H2AK5, H2BK16 and H3K27 enrichment in the promoter of *H19* gene in DLD1 WT or *HDAC2* KO cells. **d** A schematic of the *H19* promoter-luciferase construct is depicted with the locations of the SP1 binding sites and the sequences of the point mutations. **e**, **f** Dual luciferase assay of 293 cells cotransfected with the *H19* promoter reporter constructs (wild-type or mutants at three SP1 binding sites) and the shHDAC2 or HDAC2 plasmids. **g** Interaction between HDAC2 and SP1 displayed by Co-IP. **h**, **i** Expression of H19 in SP1 RNAi CRC cells. **P < 0 .01; *P < 0.05. The data are representatives and are presented as mean ± standard error of the mean of 3 assays

To understand how HDAC2 regulates the expression of H19 in CRC, we performed chromatin immunoprecipitation experiments and luciferase reporter assays. The results showed that HDAC2 can bind to the H19 promoter (Fig. 4a), and it deletion promotes histone H3K27 acetylation in the DLD1 *HDAC2* KO cells (Fig. 4b). In addition, ChIP experiments confirmed that the level of acetylated histone H3K27 at the *H19* promoter increases in DLD1 *HDAC2* KO cells (Fig. 4c). Collectively, these results indicate that HDAC2 catalyzes H3K27 deacetylation in *H19* promoter.

To explore transcription factors involved in the regulation of HDAC2-mediated H19 expression, we analyzed the DNA sequence of the *H19* promoter (Fig. S4a). Three binding sites for the transcription factor SP1 were found in *H19* promoter, and its reporters with mutated SP1 recognition site were constructed (Fig. 4d). the reporter gene assay showed that HDAC2 attenuates H19 transcriptional activation (Fig. 4e and f). Furthermore, a direction interaction between HDAC2 and SP1 was confirmed by immunoprecipitation in both DLD1 cells (Fig. 4g left) and SW480 cells (Fig. 4g right). In addition, *SP1* knockdown by siRNA in DLD1 and SW480 cells significantly increases H19 expression (Fig. 4h and i), cell migration (Fig. S4b and c), and modulates the expression of EMT markers including decreased E-cadherin, increased fibronectin and ITGA5 (Fig. S4d). Together, these results suggest that HDAC2 inhibits the expression of H19 and EMT through SP1 binding-dependent H3K27 deacetylation.

### H19 promotes EMT by upregulating MMP14

**Fig. 5:**
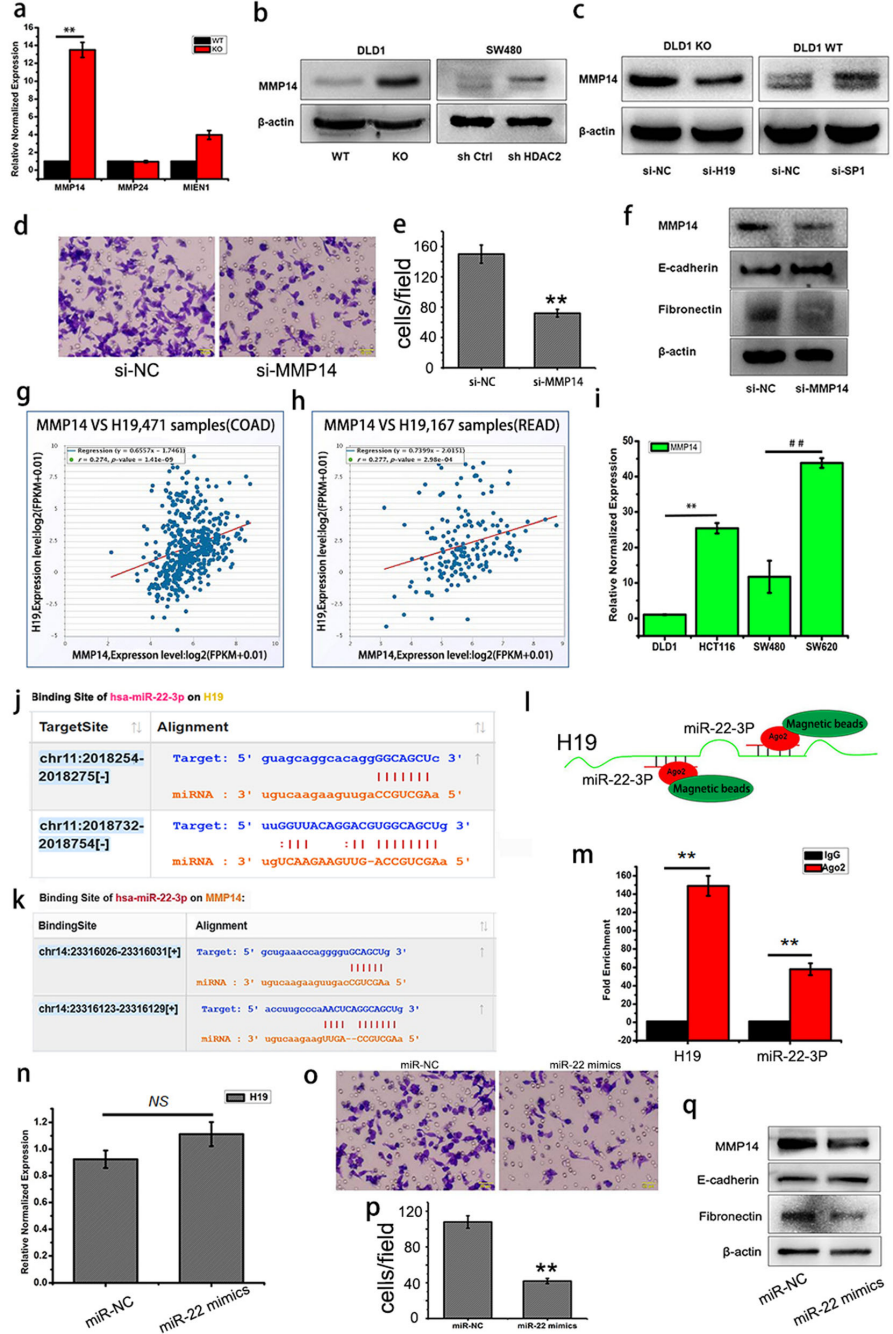
H19 promotes EMT by sponging miR-22 and upregulating MMP14. **a** Expression of MMPs in DLD1 *HDAC2* KO cells. **b**, **c** Detection of MMP14 by Western blot. **d**, **e** Migration ability of DLD1 *HDAC2* KO MMP14 RNAi cells detected by transwell. **f** Detection of EMT markers by Western blot in DLD1 *HDAC2* KO MMP14 RNAi cells. **g**, **h** The correction between H19 and MMP14 expression in colorectal cancer samples. Data were collected from TCGA, including 471 cases of COAD and 167 cases of READ. **i** Expression of MMP14 in CRC cells. **j** Schematic diagrams of the mutual interactions between miRNA- 22-3P and H19. **k** Schematic diagrams of the mutual interactions between miRNA-22-3P and 3’UTR of MMP14. **l**, **m** The interactions between miRNA- 22-3P and H19 detected by anti-Ago2 RIP in DLD1 *HDAC2* KO cells. **n** Expression of H19 in DLD1 *HDAC2* KO cells transfected with miRNA- 22-3P mimincs. **o**, **p** Migration of DLD1 *HDAC2* KO cells transfected with miRNA- 22-3P mimincs detected by transwell. **q** Detection of EMT markers and MMP14 by Western blot in DLD1 *HDAC2* KO cells transfected with miRNA- 22-3P mimincs. **P < 0 .01; *P < 0.05. The data are representatives and are presented as mean ± standard error of the mean of 3 assays

H19 may promote EMT by sponging miRNA-138 and miR-200a, and then upregulation of their downstream targets Vimentin,ZEB1 and ZEB2 in colorectal cancer [29]. However, we did not detect an increase in *Vimentin, ZEB1* and *ZEB2* in the DLD1 *HDAC2* KO cells in the microarray data. Instead, we noticed significant changes in several MMPs, which are well known factors of EMT [30, 31]. Q-PCR confirmed that MMP14 mRNA is the most up-regulated MMPs in DLD1 *HDAC2* KO cells (Fig. 5a), and it protein expression also is increase in *HDAC2* KO and knock down cells (Fig. 5b) and in DLD1 WT cells with *SP1* knockdown (Fig. 5c right), while decrease in DLD1 *HDAC2* KO cells with *H19* knockdown (Fig. 5c left). Further, EMT was reversed in DLD1 *HDAC2* KO cells by MMP14 knockdown using siRNA (Fig. 5d,e and f). These findings indicate that H19 promotes EMT by upregulating MMP14 in CRC cells.

Furthermore, the TCGA data also showed that the expression of H19 is positively correlated with the expression of MMP14 in CRC (Fig. 5g and h). The expression of MMP14 is also higher in CRC cells with higher H19 expression (Fig. 5i). In addition, two recognition sites of microRNA-22-3P were identified in H19(Fig. 5j) and in the 3′-UTR region of *MMP14*(Fig. 5k)respectively. These findings support that H19 may up-regulate MMP14 by sponging miR-22-3P and then release its inhibitory effect on MMP14. As expected, anti-Ago2 RNA Binding Protein Immunoprecipitation (RIP) assay (Fig. 5l) showed direct binding of miR-22-3P to lncRNA H19(Fig. 5m). In addition, transfection of miR-22-3P mimics significantly decreased the expression of MMP14 (Fig. 5q) and EMT (Fig. 5o-q) in DLD1 *HDAC2* KO cells, without affecting the expression of H19(Fig. 5n). These results indicate that H19 promotes EMT by binding to miR-22-3P and upregulating the expression of MMP14.

### Loss of HDAC2 expression promotes colorectal cancer metastasis in vivo

**Fig. 6:**
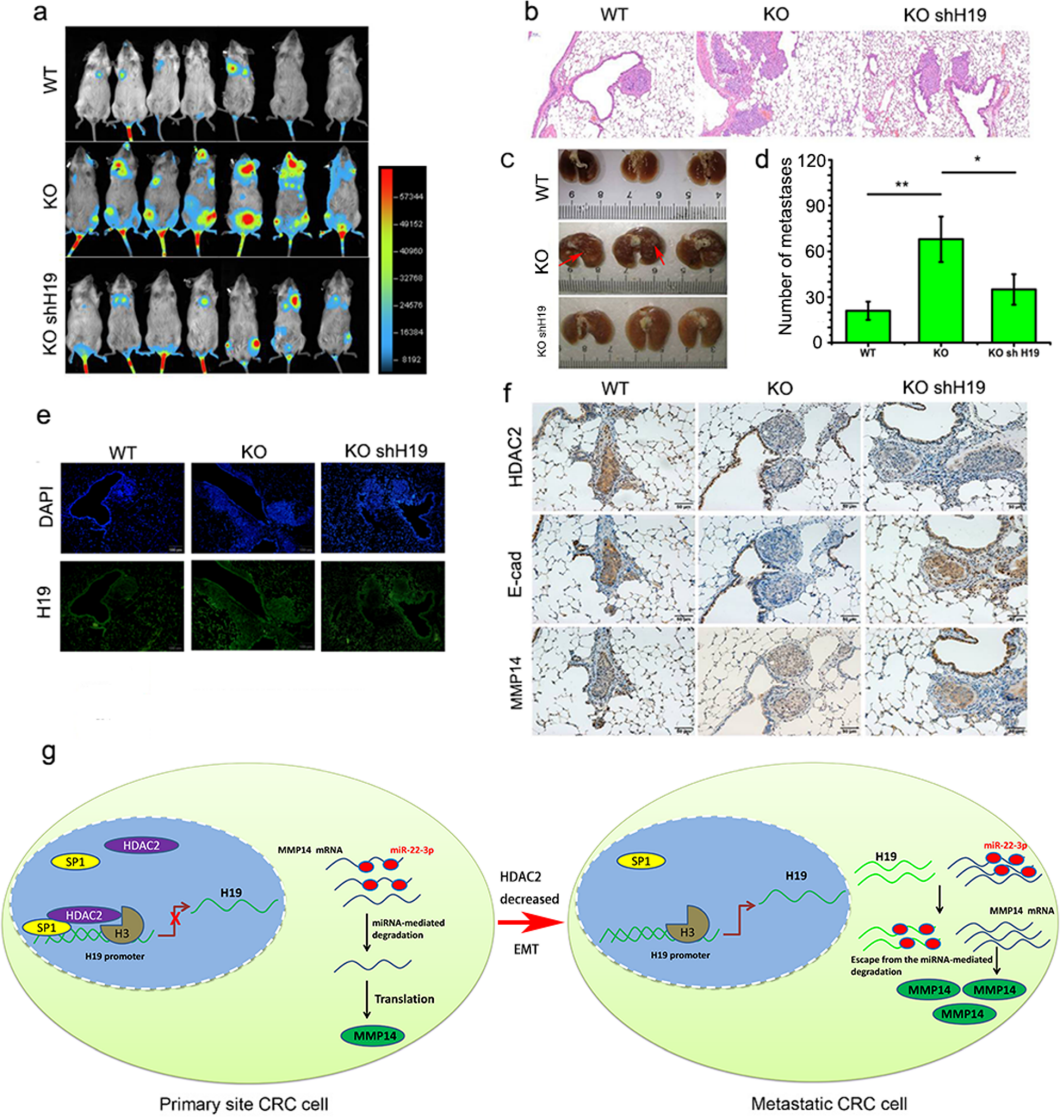
Low HDAC2 expression promotes colorectal cancer metastasis in vivo. **a** Metastatic tumors were detected and photographed by a bioluminescent in vivo imager, *n* = 6. **b** Hematoxylin and eosin-stained images of mouse lung tissues. **c** Photos of mice lungs gained from WT, KO and KO shH19 group. **d** The average number of metastatic nodules in the lungs. **e** The expression of H19 in metastatic lung nodules determined by FISH. **f** The expression of HDAC2, E-cadherin and MMP14 determined by IHC staining in metastatic nodules in the lungs. **g** Schematic depicting mechanism of HDAC2 regulating CRC metastasis. **P < 0 .01; *P < 0.05. The data are presented as mean ± standard error of the mean

To further evaluate the role of HDAC2 in CRC metastasis in vivo, we inoculated nude mice with luciferase-labeled DLD1 WT, *HDAC2* KO and *HDAC2* KO shH19 cells via the caudal vein. The results showed that, compared with the WT group, the luciferase signal intensities (Fig. 6a) and lung metastases (Fig. 6b, c and d) are significantly increased in the *HDAC2* KO group while impaired by *H19* knockdown. Furthermore, increased H19 and MMP14, but decreased E-cadherin were detected in the lung metastases of *HDAC2* KO group (Fig. 6e and f), while reversed by *H19* knockdown (Fig. 6e and f). Additionally, the results of inoculation with luciferase-labeled SW480 and SW620 cells (a high metastatic potential of CRC cell line) in nude mice also showed that lung metastases (Fig. S5a-c) increase in the SW620 group, and with a decrease of HDAC2 and E-cadherin expression, but increase of MMP14 expression (Fig. S5d). Collectively, these results support that reduced HDAC2 expression promotes EMT-mediated CRC metastasis in vivo by upregulating H19 and MMP14.

## Discussion

Here, we reported a novel function of HDAC2 in suppressing CRC metastasis. The expression of HDAC2 was reduced in CRC metastasis tissues, and reduce expression is associated with poor survival of CRC patients. Loss of HDAC2 expression promoted EMT-mediated CRC metastasis via the H19/MMP14 axis (Fig. 6g). HDACs have been reported to be highly expressed in many tumors, and the development of cancer drugs targeting HDACs has been carried out for many years [32]. Unfortunately, clinical trials have shown that HDAC inhibitors, as single agent, do not benefit patients with solid tumors including colorectal cancer [33]. Several HDAC inhibitors were reported to induce EMT and metastasis [24, 34]. These findings are consistent with our results, and support that some HDACs might have tumor suppressive functions. Therefore, more selective HDACi are likely needed in order for further clinical development in solid tumors.

HDACs are extended families of proteins regulating gene expression and cell physiology through many targets. We focused our studies on class I HDACs in CRC metastasis upon the finding of a negative correlation of HDAC2 expression and CRC patient survival. HDACs have been reported to regulate EMT and cancer metastasis either positively or negatively. Typical EMT markers include cell adhesion molecules such as E-cadherin, Vimentin, N-cadherin, ITGA5and Fibronectin, and transcription factors such as Snail1/2, ZEB1/2, and ZO-1 [4, 35].Most HDACs were found to promote EMT and metastasis by binding to EMT-related transcription factors and directly inhibit the expression of epithelial markers such as E-cadherin. In this study, we also found a decrease of E-cadherin and increase of Fibronectin and ITGA5, but not an increase of Vimentin, ZEB1 and ZEB2 in the DLD1 HDAC2 KO microarray data. The reason is that not all types of cells or tissues share the same EMT markers, and this may be a feature of EMT induced by HDAC2 loss in CRC cells. Furthermore, we found a novel function of HDAC2 in suppressing CRC metastasis. HDAC2 regulates EMT and metastasis indirectly by inhibiting lncRNA H19 and MMP14. These findings reinforce context-dependent role of HDACs, and highlight the critical need to understand their cancer type specific roles.

LncRNA H19 is the first described human lncRNA and implicated in cancer initiation, progression and metastasis [36]. Especially, H19 has been reported to up-regulated in CRC tissues compared with non-tumor tissues and H19 overexpression is closely associated with poor survival of CRC patients [37–39]. Besides, several studies showed that H19 was upregulated in metastatic cancer tissues and could promote cancer metastasis, including colorectal cancer [29, 40, 41]. However, the mechanistic insights of deregulated H19 expression in cancer are limited [42, 43]. Our study firstly reported an inverse correlation between HDAC2 and H19 expression levels in CRC and verified H19 as a possible downstream target of HDAC2 in promoting EMT and MMP14 expression. H19 promotes EMT and cancer metastasis through a variety of mechanisms. H19 promoted glioma cell invasion by deriving miR-675 [27]. H19 promoted EMT by sponging miRNAs in colorectal cancer [29]. H19 increased bladder cancer metastasis by binding to EZH2 and inhibiting E-cadherin expression [28]. In this study, we found that H19 promotes EMT and CRC metastasis by sponging miR-22-3P and upregulating MMP14. These findings support that HDACs and LncRNAs form gene expression regulatory networks to control CRC development. Actually, microarray data showed *HDAC2* deletion affects the expression of thousands of genes in CRC cells. Apart from the lncRNA H19 signaling axis, HDAC2 maybe still has other underlying molecular mechanisms in the regulation of CRC EMT and metastasis, and need more experiment to verify it in the future.

Several evidences showed that the expression of HDAC2 in CRC tissues is up-regulated compared with normal colon tissues [44–46]. HDAC2 up-regulation has been reported to be a novel and important early event in CRC [44]. These results indicate an oncogenic role of HDAC2 in CRC. However, in this study, we found HDAC2 expression decreases in metastastic CRC (compared with primary site CRC), and reduced HDAC2 expression is associated with poor survival of CRC patients. Actually, the occurrence and metastasis of cancer are two independent events. We think HDAC2 plays a dual role in the development of CRC, that is promoting the initiation of CRC but suppressing CRC metastasis. Explore the origins of dynamic HDAC2 expression in CRC is a valuable question in the future.

## Conclusion

In this study, we elucidated a novel role of HDAC2 in suppressing CRC metastasis. HDAC2 expression was reduced in CRC metastasis and reduced expression predicts poor outcome in CRC patients. Mechanistically, HDAC2 inhibits EMT and CRC metastasis by binding to SP1 to suppress H19/MMP14 expression. The HDAC2/ H19/MMP14 axis might provide novel targets for developing anti-metastasis agents in CRC patients.

## Supplementary Information

**Additional file 1.**

**Additional file 2.**

**Additional file 3.**

## Data Availability

The datasets generated and/or analyzed during the current study are available from the supplementary information or the corresponding author upon reasonable request.

## Abbreviations

CRC: Colorectal cancer
EMT: Epithelial to mesenchymal transition
LncRNA: Long non-coding RNA
HDAC2: Histone deacetylases

## References

[1] Siegel RL, Miller KD, Jemal A (2018). Cancer statistics, 2018. CA Cancer J Clin.

[2] Siegel RL, Miller KD, Fedewa SA (2017). Colorectal cancer statistics, 2017. CA Cancer J Clin.

[3] Lambert AW, Pattabiraman DR, Weinberg RA (2017). Emerging biological principles of metastasis. Cell.

[4] Lamouille S, Xu J, Derynck R (2014). Molecular mechanisms of epithelial-mesenchymal transition. Nat Rev Mol Cell Biol.

[5] Nieto MA, Huang RY, Jackson RA (2016). Emt: 2016. Cell.

[6] Heerboth S, Housman G, Leary M (2015). EMT and tumor metastasis. Clin Transl Med.

[7] Davis FM, Stewart TA, Thompson EW (2014). Targeting EMT in cancer: opportunities for pharmacological intervention. Trends Pharmacol Sci.

[8] Li Y, Seto E (2016). HDACs and HDAC inhibitors in cancer development and therapy. Cold Spring Harb Perspect Med.

[9] Sudo T, Mimori K, Nishida N (2011). Histone deacetylase 1 expression in gastric cancer. Oncol Rep.

[10] Oehme I, Deubzer HE, Wegener D (2009). Histone deacetylase 8 in neuroblastoma tumorigenesis. Clin Cancer Res.

[11] Mithraprabhu S, Kalff A, Chow A (2014). Dysregulated class I histone deacetylases are indicators of poor prognosis in multiple myeloma. Epigenetics.

[12] Stark M, Hayward N (2007). Genome-wide loss of heterozygosity and copy number analysis in melanoma using high-density single-nucleotide polymorphism arrays. Cancer Res.

[13] Taylor BS, DeCarolis PL, Angeles CV (2011). Frequent alterations and epigenetic silencing of differentiation pathway genes in structurally rearranged liposarcomas. Cancer Discov.

[14] Osada H, Tatematsu Y, Saito H (2004). Reduced expression of class II histone deacetylase genes is associated with poor prognosis in lung cancer patients. Int J Cancer.

[15] Jin Z, Jiang W, Jiao F (2014). Decreased expression of histone deacetylase 10 predicts poor prognosis of gastric cancer patients. Int J Clin Exp Pathol.

[16] Lv Z, Weng X, Du C (2016). Downregulation of HDAC6 promotes angiogenesis in hepatocellular carcinoma cells and predicts poor prognosis in liver transplantation patients. Mol Carcinog.

[17] Ropero S, Fraga MF, Ballestar E (2006). A truncating mutation of HDAC2 in human cancers confers resistance to histone deacetylase inhibition. Nat Genet.

[18] Ropero S, Ballestar E, Alaminos M (2008). Transforming pathways unleashed by a HDAC2 mutation in human cancer. Oncogene.

[19] He J, Shen S, Lu W (2016). HDAC1 promoted migration and invasion binding with TCF12 by promoting EMT progress in gallbladder cancer. Oncotarget.

[20] Aghdassi A, Sendler M, Guenther A (2012). Recruitment of histone deacetylases HDAC1 and HDAC2 by the transcriptional repressor ZEB1 downregulates E-cadherin expression in pancreatic cancer. Gut.

[21] Byles V, Zhu L, Lovaas JD (2012). SIRT1 induces EMT by cooperating with EMT transcription factors and enhances prostate cancer cell migration and metastasis. Oncogene.

[22] Zeng H, Qu J, Jin N (2016). Feedback activation of leukemia inhibitory factor receptor limits response to histone deacetylase inhibitors in breast cancer. Cancer Cell.

[23] Lin KT, Wang YW, Chen CT (2012). HDAC inhibitors augmented cell migration and metastasis through induction of PKCs leading to identification of low toxicity modalities for combination cancer therapy. Clin Cancer Res.

[24] Ji M, Lee EJ, Kim KB (2015). HDAC inhibitors induce epithelial-mesenchymal transition in colon carcinoma cells. Oncol Rep.

[25] Liu Y, Li S, Zhang H (2012). A one-step cloning method for the construction of somatic cell gene targeting vectors: application to production of human knockout cell lines. BMC Biotechnol.

[26] Lin CW, Lin PY, Yang PC (2016). Noncoding RNAs in tumor epithelial-to-mesenchymal transition. Stem Cells Int.

[27] Shi Y, Wang Y, Luan W (2014). Long non-coding RNA H19 promotes glioma cell invasion by deriving miR-675. PLoS One.

[28] Luo M, Li Z, Wang W (2013). Long non-coding RNA H19 increases bladder cancer metastasis by associating with EZH2 and inhibiting E-cadherin expression. Cancer Lett.

[29] Liang WC, Fu WM, Wong CW (2015). The lncRNA H19 promotes epithelial to mesenchymal transition by functioning as miRNA sponges in colorectal cancer. Oncotarget.

[30] Yang CC, Zhu LF, Xu XH (2013). Membrane type 1 matrix metalloproteinase induces an epithelial to mesenchymal transition and cancer stem cell-like properties in SCC9 cells. BMC Cancer.

[31] Yan T, Lin Z, Jiang J (2015). MMP14 regulates cell migration and invasion through epithelial-mesenchymal transition in nasopharyngeal carcinoma. Am J Transl Res.

[32] Montezuma D, Henrique RM, Jeronimo C (2015). Altered expression of histone deacetylases in cancer. Crit Rev Oncog.

[33] McClure JJ, Li X, Chou CJ (2018). Advances and challenges of HDAC inhibitors in cancer therapeutics. Adv Cancer Res.

[34] Wang J, Xu MQ, Jiang XL (2018). Histone deacetylase inhibitor SAHA-induced epithelial-mesenchymal transition by upregulating slug in lung cancer cells. Anti-Cancer Drugs.

[35] Mittal V (2018). Epithelial mesenchymal transition in tumor metastasis. Annu Rev Pathol.

[36] Raveh E, Matouk IJ, Gilon M (2015). The H19 long non-coding RNA in cancer initiation, progression and metastasis - a proposed unifying theory. Mol Cancer.

[37] Ding D, Li C, Zhao T (2018). LncRNA H19/miR-29b-3p/PGRN Axis promoted epithelial-mesenchymal transition of colorectal cancer cells by acting on Wnt signaling. Mol Cells.

[38] Zhong ME, Chen Y, Zhang G (2019). LncRNA H19 regulates PI3K-Akt signal pathway by functioning as a ceRNA and predicts poor prognosis in colorectal cancer: integrative analysis of dysregulated ncRNA-associated ceRNA network. Cancer Cell Int.

[39] Xu Y, Wang Z, Jiang X (2017). Overexpression of long noncoding RNA H19 indicates a poor prognosis for cholangiocarcinoma and promotes cell migration and invasion by affecting epithelial-mesenchymal transition. Biomed Pharmacother.

[40] Zhang Y, Huang W, Yuan Y (2020). Long non-coding RNA H19 promotes colorectal cancer metastasis via binding to hnRNPA2B1. J Exp Clin Cancer Res.

[41] Chen Y, Yu X, Xu Y (2017). Identification of dysregulated lncRNAs profiling and metastasis-associated lncRNAs in colorectal cancer by genome-wide analysis. Cancer Med.

[42] Dugimont T, Montpellier C, Adriaenssens E (1998). The H19 TATA-less promoter is efficiently repressed by wild-type tumor suppressor gene product p53. Oncogene.

[43] Cui J, Mo J, Luo M (2015). c-Myc-activated long non-coding RNA H19 downregulates miR-107 and promotes cell cycle progression of non-small cell lung cancer. Int J Clin Exp Pathol.

[44] Stypula-Cyrus Y, Damania D, Kunte DP (2013). HDAC up-regulation in early colon field carcinogenesis is involved in cell tumorigenicity through regulation of chromatin structure. PLoS One.

[45] Zhu P, Martin E, Mengwasser J (2004). Induction of HDAC2 expression upon loss of APC in colorectal tumorigenesis. Cancer Cell.

[46] Mao QD, Zhang W, Zhao K (2017). MicroRNA-455 suppresses the oncogenic function of HDAC2 in human colorectal cancer. Braz J Med Biol Res.

