# Supplementary material for: HDAC2 inhibits EMT-mediated cancer metastasis by downregulating the long noncoding RNA H19 in colorectal cancer
Source: J Exp Clin Cancer Res. 2020 Dec 2;39:270. doi: 10.1186/s13046-020-01783-9 (PMC7709355; doi:10.1186/s13046-020-01783-9)
Supplement: Supplementary file 1 — Additional file 1. [file 13046_2020_1783_MOESM1_ESM.doc]

**Supplementary information**

**Materials and methods：**

**Real-time cell migration assay (RTCA)**

The migration rates of different cancer cells were monitored by using an xCELLigence Real-Time Cell Analyzer (RTCA) DP Instrument (ACEA Biosciences, Hangzhou, China). This platform is able to measure cell migration and invasion in real time by monitoring changes in electrical impedance. The electrical impedance, which is defined as cell index values, is increased when cells pass through a porous membrane and contact and adhere to electronic sensors on the underside of the membrane (Bird C. and Kirstein S. Real-time, label-free monitoring of cellular invasion and migration with the xCELLigence system. Nature Methods, 2009, 6). Briefly, 165 µl of RPMI 1640 culture medium with 10% FBS was loaded onto the lower chamber of a cell invasion and migration plate (CIM-Plate 16; Roche). The upper chamber was then fitted on the lower chamber and loaded with 30 µl of RPMI 1640 serum-free culture medium. After 1 h of equilibration, to measure the baseline of CIM-Plate 16, 100 μl mixed serum-free cell suspensions (8 × 10^4^ cells) were loaded on each well of the upper chamber. The CIM-Plate was subsequently placed on the RTCA analyzer in a 37°C incubator. Cell migration was recorded every 15 min (100 sweeps at 15-min intervals) using the cell index provided by the RTCA DP instrument. Four independent reactions were assayed for each condition.

**RNA isolation and real-time reverse transcription PCR (qRT-PCR)**

Total RNA was isolated using a high-purity total RNA extraction kit (Biotech). First-strand cDNA was generated using a reverse transcription kit (Takara). Real-time PCR was performed in a CFX-96 Real-Time PCR Detection System (Bio-Rad) using SYBR Green (Takara, Dalian, China) and the gene-specific primers shown in the primer sequences section below. The relative expression of the RNAs was calculated using the comparative Ct method. The levels of β-actin mRNA were used as an endogenous control to normalize for differences in the amount of total RNA.

| **Primer names** | **Forward (5’-3’)** | **Reverse (5’-3’)** |
| --- | --- | --- |
| **HDAC2** | GCTATTCCAGAAGATGCTGTTC | GTTGCTGAGCTGTTCTGATTTG |
| **H19** | CTTCTGGGCTCAAGTGATCCT | TTGTGCCATGAGACTCCATCAG |
| **E-cadherin** | TGCTCTTCCAGGAACCTCTGT | GTAAGCGATGGCGGCATTGTA |
| **MMP14** | TCCAGCAACTTTATGGGGGT | TTCCCGTCACAGATGTTGGG |
| **MMP24** | AGGATCCACTCACCATCGGA | GCCAGAACCAGCGATCCTTA |
| **MIEN1** | GTGAAGGAGCAGTATCCGGG | GGTCCTCCCAACGCTGTAAA |
| **β-actin** | CTTAGTTGCGTTACACCCTTTCTTG | ACTGCTGTCACCTTCACCGTTC |

**Protein extraction and Western blot**

Western blotting was performed by the following steps. Briefly, cells were washed with ice-cold PBS, lysed in RIPA buffer containing cocktail and then centrifuged for 10 min at 12000 rpm at 4°C. Protein concentrations were measured by using a BCA assay reagent. Total cell lysates were prepared in 5× SDS loading buffer. Identical quantities of proteins were separated by SDS-PAGE and transferred onto PVDF membranes. After incubation with antibodies specific for HDAC2（CST 57156）, E-cadherin（CST 14472）, fibronectin（Proteintech Cat 15613-1-AP）, ITGα5（Proteintech Cat 10569-1-AP）, MMP14（Abcam ab51074）, SP1（CST 9389） or β-actin（CST 3700）, the blots were incubated with goat anti-rabbit IgG or goat anti-mouse IgG and detected using a chemiluminescence detection system (Bio-Rad, USA). β-Actin was used as a loading control for Western blots.

**Immunofluorescence**

DLD1 and DLD1HDAC2 KO cells were cultured and fixed on 12×12 mm glass slides. After first being incubated with antibodies specific for E-cadherin or fibronectin and then goat anti-rat IgG (Alexa Fluor 594, Invitrogen), goat anti-mouse IgG (Alexa Fluor 488, Invitrogen) or goat anti-rabbit IgG (Alexa Fluor 594, Invitrogen), the slides were mounted by adding DAPI (0.1 mg/ml) and examined with fluorescence microscopy (Olympus).

**Immunofluorescence for actin-containing structures**

DLD1 and DLD1HDAC2 KO cells were cultured in 24-well plate and fixed in 4% paraformaldehyde for 20 mins. After that, cells were washed with 0.1% Triton X-100(twice, 5 mins each time) and incubated with Actin-Tracker Red-555 (phalloidin- Alexa Fluor 555, Beyotime, C2203S) for an hour. At last, the pictures were taken with a fluorescence microscope (Olympus).

**Results：**

**
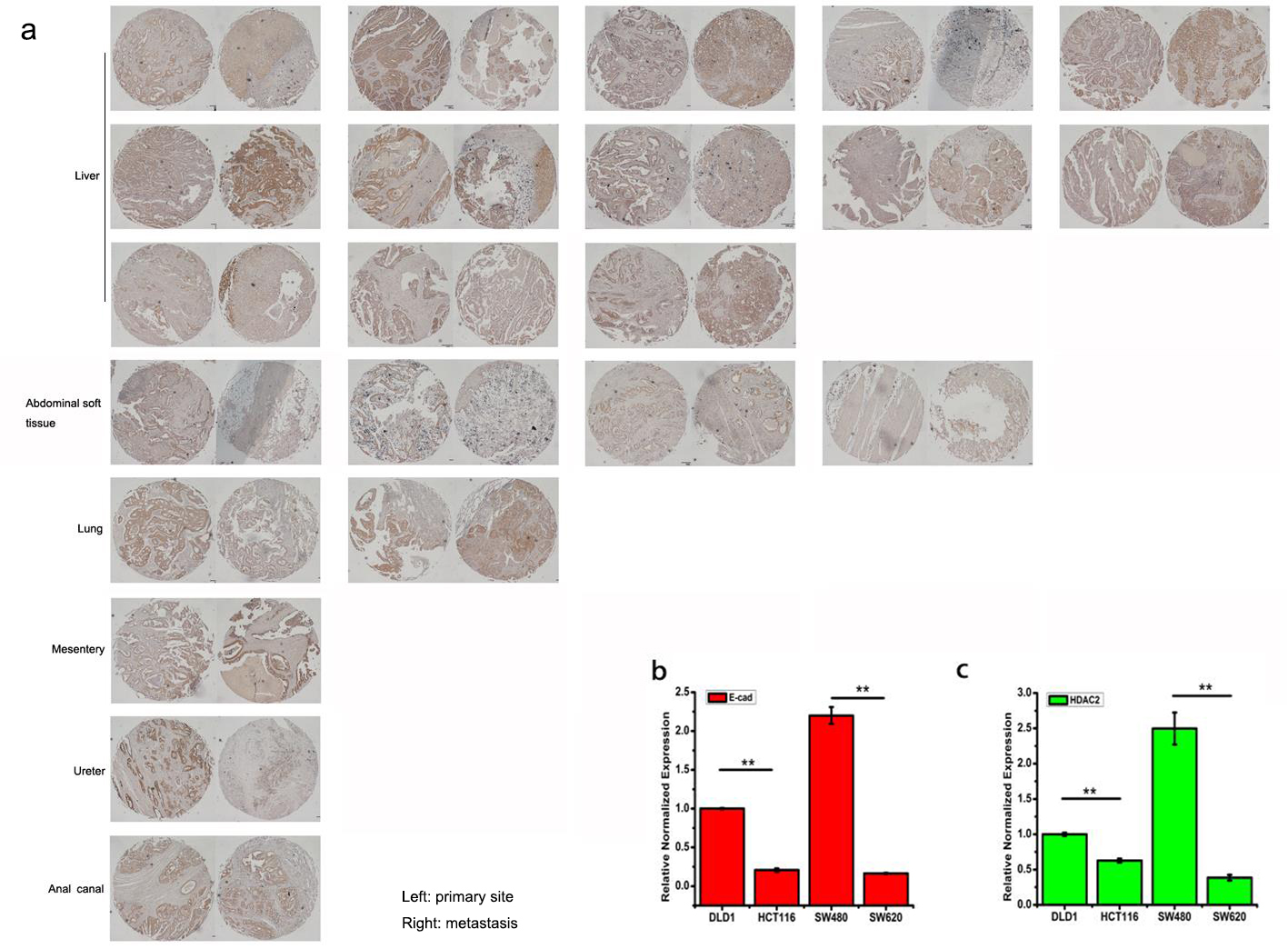
**

Figure S1.

a. Expression of HDAC2 in 22 paired-CRC samples detected by IHC.

b,c Expression of E-cadherin and HDAC2 in CRC cells.


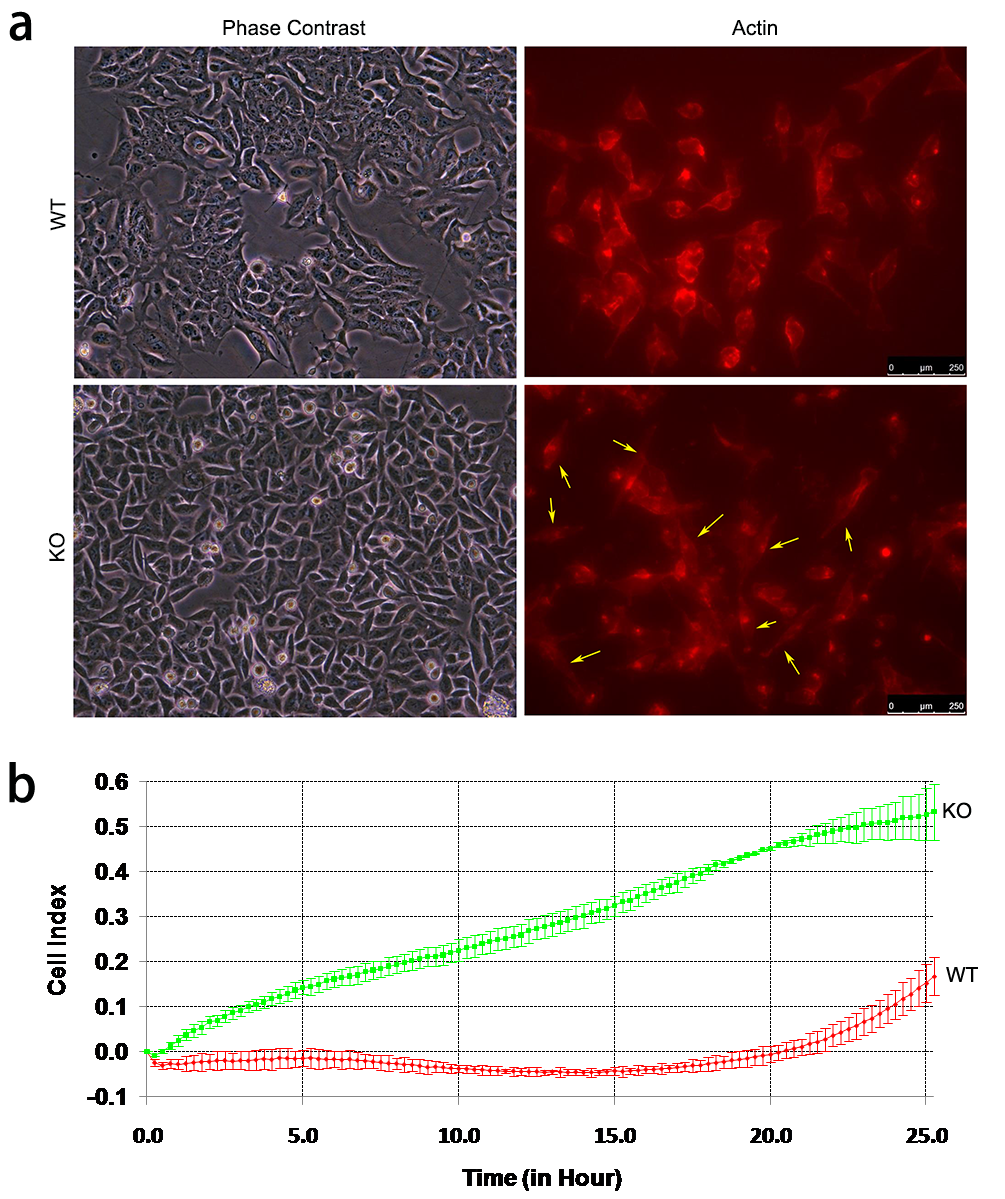


Figure S2.

a. Morphological changes in DLD1 HDAC2 KO cells(100x).

b.Migration ability of DLD1 HDAC2 KO cells detected by RTCA


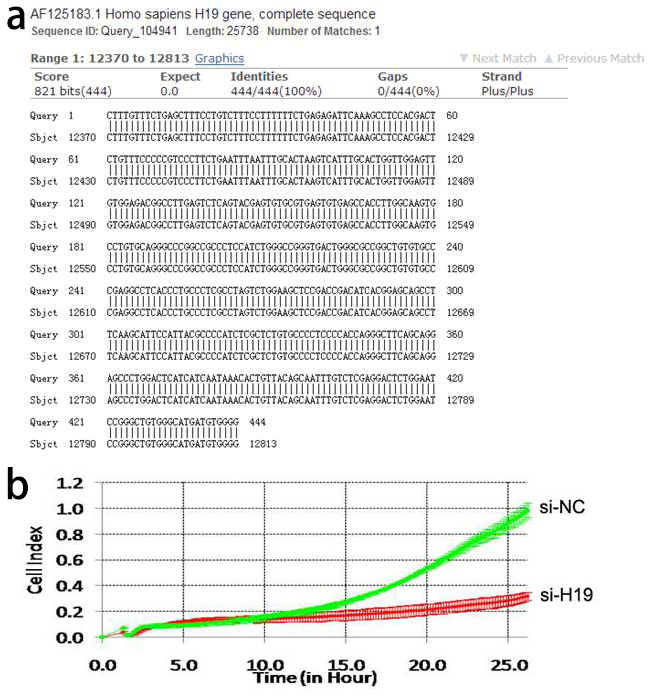


Figure S3.

a. N333410 was 100% identified to H19 gene sequence.

b.Migration ability of si-H19 cells were detected by RTCA.


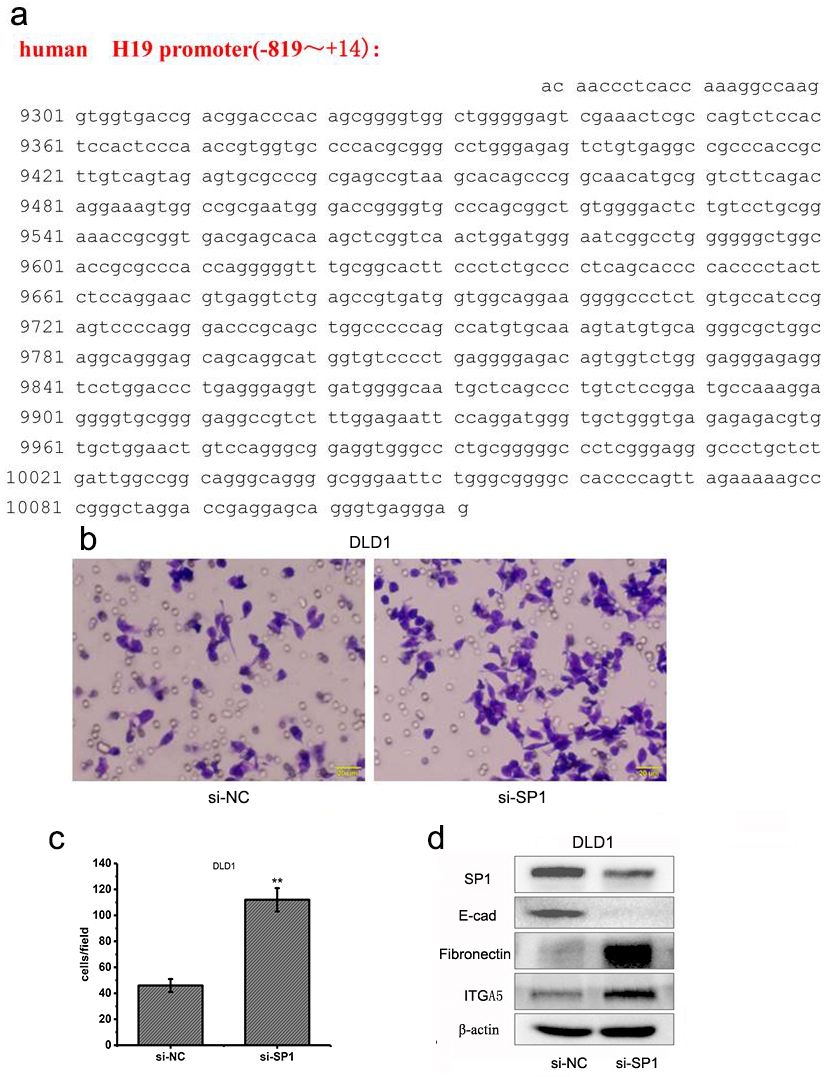


Figure S4.

a. DNA sequence of human H19 gene promoter.

b.c Migration ability of DLD1 SP1 RNAi cells analyzed by transwell.

d. Detection of EMT markers by Western Blot.


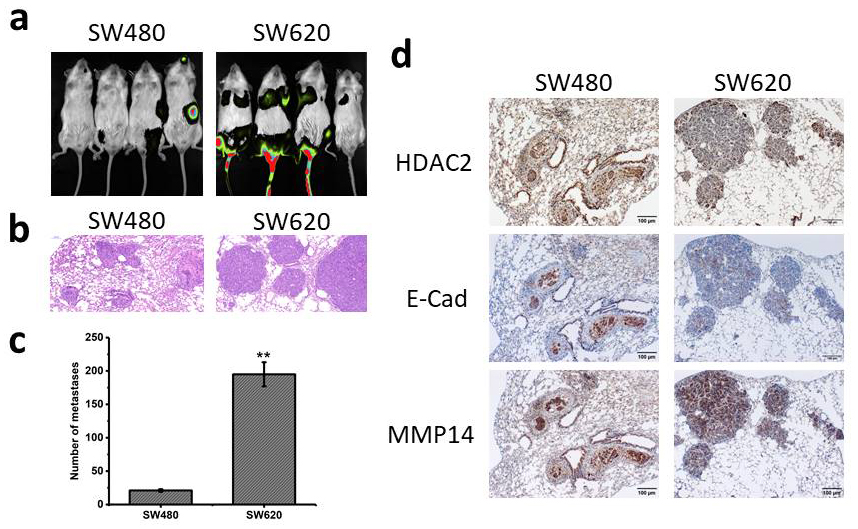


Figure S5.

a. Metastatic tumors were detected and photographed by a bioluminescent in vivo imager, n=4.

b. Hematoxylin and eosin-stained images of mouse lung tissues.

c. The average number of metastatic nodules in the lungs.

d. Expression of HDAC2, E-cadherin and MMP14 determined by IHC staining in metastatic nodules in the lungs.
